# Embedding virtual reality social skills training into return‐to‐work care for depression: A single‐arm feasibility pilot with exploratory autistic‐trait moderation

**DOI:** 10.1002/pcn5.70289

**Published:** 2026-02-02

**Authors:** Yutaro Akaki, Yasuhisa Nakamura, Yuko Nakanishi, Yuji Okuyama, Kiyoshi Fujita

**Affiliations:** ^1^ Seishinkai Medical Corporation Fujita Mental Care Satellite Tokushige‐Kita Re‐Work Center Nagoya Aichi Japan; ^2^ Department of Rehabilitation, Faculty of Health Sciences Nihon Fukushi University Handa Aichi Japan; ^3^ Department of Psychiatry, Okehazama Hospital Fujita Kokoro Care Center Toyoake Aichi Japan

**Keywords:** depression, psychiatric day‐care, return to work, virtual reality (VR)

## Abstract

**Aim:**

To assess the feasibility of embedding virtual reality‐based social skills training (SST‐VR) into a return‐to‐work day‐care program for major depressive disorder (MDD) and to estimate pre–post change in self‐reported social skills, with exploratory moderation by autistic traits.

**Methods:**

In this single‐arm, add‐on pilot at a Japanese psychiatric day‐care Re‐Work center, 20 adults with MDD (18 men) received six 90‐min SST‐VR sessions every 2 weeks over approximately 3 months plus the standard Re‐Work program. The primary outcome was Kikuchi's Social Skills Scale–18 (KiSS‐18); secondary outcomes were social adaptation, social anxiety, and depressive symptoms (self‐report). A linear mixed‐effects model tested the effects of Time (pre vs post), baseline Autism‐Spectrum Quotient (AQ; mean‐centered), and their interaction. Feasibility was assessed via attendance and attrition.

**Results:**

Attendance was 94.2% with no attrition. KiSS‐18 increased from 49.7 ± 10.6 to 53.5 ± 12.4 (*p* = 0.028; *r* = 0.56). The model showed significant effects of Time (*χ*²(1) = 8.11, *p* = 0.004) and Time × AQ (*χ*²(1) = 4.46, *p* = 0.035), suggesting smaller gains at higher AQ (exploratory; restricted AQ range). Emotional processing and stress management subscales improved; secondary outcomes showed no significant change. Bootstrap analyses were consistent with the mixed‐model findings.

**Conclusion:**

SST‐VR was feasible. Because the single‐arm add‐on design cannot isolate SST‐VR–specific effects from concurrent care and nonspecific influences, the KiSS‐18 change is hypothesis‐generating. Controlled comparisons with Re‐Work alone in larger, more diverse samples should test efficacy, include objective behavioral and vocational outcomes, and prospectively evaluate moderation.

## INTRODUCTION

Depression is a major contributor to the global burden of disease, and many patients continue to experience impaired social functioning even after symptomatic remission.^1^, ^2^ Because successful return to work and sustained employment depend on day‐to‐day communication and adaptation in the workplace, persistent social difficulties can undermine recovery and increase the risk of recurrent sick leave.^3^, ^4^, ^5^ Accordingly, return‐to‐work care may benefit from interventions that address social functioning directly, alongside symptom‐focused treatment.

In Japan, Re‐Work programs have been widely implemented as structured return‐to‐work day‐care services. Evidence indicates that residual social anxiety and lower social adaptation predict poorer work maintenance after sick leave.^6^, ^7^ However, opportunities for structured, repeated practice of workplace‐relevant interpersonal skills remain limited in routine care and in many standard return‐to‐work interventions, which often emphasize symptom management, cognitive remediation, and occupational training.^3^, ^8^, ^9^ This gap motivates the development of practical ways to embed social skills practice within return‐to‐work settings.

Social skills training (SST) is a structured behavioral intervention that promotes skill acquisition through modeling, role‐play, and feedback.^10^, ^11^ In depression, SST has been reported to improve interpersonal functioning and workplace adaptation.^12^, ^13^ At the same time, conventional SST can struggle to reproduce high‐stress workplace situations in a consistent way, and the experience may vary across participants and sessions.^10^, ^14^ These constraints can make practice less standardized and may limit opportunities to reflect on shared challenges across a group.

Immersive virtual reality (VR) offers a complementary approach by allowing individuals to rehearse realistic social situations repeatedly in a safe and controllable environment.^15^, ^16^, ^17^ By integrating visual and auditory cues, VR can strengthen the sense of presence and may facilitate the recognition of social signals compared with traditional role‐play.^18^, ^19^, ^20^ Standardized VR scenarios can also provide a common reference point for group discussion, potentially enriching reflection on strategies and perspectives.^21^, ^22^

Evidence from schizophrenia and social anxiety disorder suggests that VR‐delivered SST can reduce interpersonal anxiety and improve social skills.^22^, ^23^, ^24^, ^25^ In contrast, studies of VR‐based SST (SST‐VR) for depression in Japan are scarce, and integration into return‐to‐work day‐care programs has rarely been evaluated. It is also uncertain whether autistic traits, assessed by the Autism‐Spectrum Quotient (AQ), influence pre–post change in social skills when SST‐VR is delivered as an add‐on to return‐to‐work care. Because autistic traits may shape social learning processes, any moderation—if present—could inform stratification or tailoring in future trials.^26^, ^27^

Accordingly, this study piloted the integration of SST‐VR into a Japanese return‐to‐work day‐care program for adults with MDD. We assessed feasibility (attendance and attrition), estimated pre–post changes in self‐reported social skills, and exploratorily examined whether baseline AQ moderated these changes to inform the design of future controlled studies.

## METHODS

### Study design

This single‐arm, add‐on feasibility pilot evaluated whether virtual reality‐based social skills training (SST‐VR) could be integrated into a standard return‐to‐work day‐care (Re‐Work) program for adults with major depressive disorder (MDD). Participants received SST‐VR in addition to usual Re‐Work care. Outcomes were assessed preintervention and post‐intervention to estimate pre–post changes, and feasibility was assessed primarily via attendance and attrition. Reporting followed the CONSORT extension for pilot and feasibility trials, with the checklist provided in the Supplement.

### Participants

**Figure 1:**
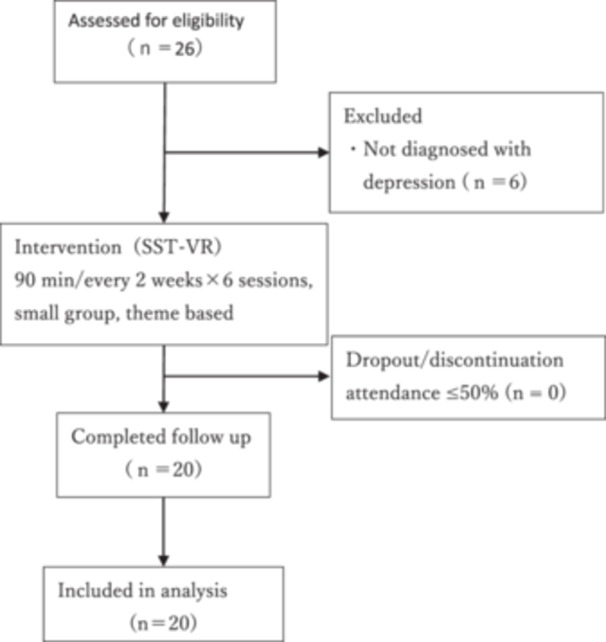
Flowchart of participant screening, enrollment, and follow‐up in the SST‐VR study.

Twenty‐six individuals were initially referred as potential participants. Eligibility required a DSM‐5 diagnosis of MDD confirmed by a psychiatrist. Participants were receiving outpatient pharmacotherapy, were clinically stable for community living, and were enrolled in the psychiatric day‐care Re‐Work program at baseline. Individuals receiving other employment support services were not eligible. After screening and written informed consent, 20 participants were enrolled and included in the analyses (reasons for nonenrollment are summarized in Figure 1). Because this was a feasibility pilot, no a priori sample size calculation was performed; the sample size was determined pragmatically based on service capacity and recruitment feasibility.

Exclusion criteria were current or lifetime bipolar I or II disorder, schizophrenia spectrum or other psychotic disorders, severe neurological disorders, current or lifetime alcohol‐ or substance‐use disorder, and any condition judged likely to interfere with participation or assessments. Baseline demographic and clinical characteristics (including sex and illness duration) are summarized in Table 1. The cohort was predominantly male (18 men and 2 women).

**Table 1:** Baseline demographic and clinical characteristics of participants (*n* = 20).

Characteristic	Value
Age, years, mean (SD)	43.5 (8.9)
Sex, *n* (men/women)	18/2
Duration of illness, months, mean (SD)	73.3 (65.2)
Employment history, years, mean (SD)	20.2 (9.4)
Attendance rate for SST‐VR, %, mean (SD)	94.2 (9.8)
Attendance rate for Re‐Work program, %, mean (SD)	90.9 (11.1)
AQ total score, mean (SD) [range]	24.5 (8.0) [8–40]
Adverse events during SST‐VR sessions, *n*	0

Abbreviations: AQ, Autism‐Spectrum Quotient; SST‐VR, virtual reality–based social skills training.

The study was conducted between September 2024 and August 2025. The study protocol was approved by the Ethics Committee of Okehazama Hospital, Fujita Kokoro Care Center (Approval No.: R06‐026). Written informed consent was obtained from all participants following a detailed explanation of the study. This study was conducted in accordance with the Declaration of Helsinki.

### Intervention

#### Setting

At the time of the intervention, all participants were attending a psychiatric day‐care return‐to‐work program (Re‐Work) that provided structured vocational rehabilitation services. The program aimed to facilitate return to work within approximately 1 year and included individual counseling and casework, as well as group programs that supported daily routines, promoted self‐reflection and insight, and developed adaptive work behaviors. SST‐VR was introduced as an adjunct component within this program to provide structured practice of workplace‐relevant interpersonal skills.

### Implementation of SST‐VR

In this study, the “FACEDUO for SST‐VR” program, jointly developed by Otsuka Pharmaceutical Co., Ltd. and Jolly Good Inc., was implemented within the existing Re‐Work program. The program uses VR head‐mounted displays and monitor‐based video presentation to support rehearsal of typical interpersonal scenarios relevant to return‐to‐work care and to provide a shared reference point for group discussion.

The intervention comprised six 90‐minute sessions delivered every 2 weeks over approximately 3 months, in small groups of 4–10 participants. The following six scenarios were selected from the FACEDUO program for their relevance to return‐to‐work support: (1) making small talk during break time; (2) asking questions when uncertain about work tasks; (3) avoiding potential sources of conflict; (4) responding appropriately to workplace feedback or warnings; (5) expressing gratitude for support received; and (6) making requests to others.

Each session included three components: experiencing the situation, identifying strategies, and practical rehearsal. Participants could engage directly using the VR device or observe the scenario via a monitor and participate in the group discussion. All participants used the VR device at least once during the intervention period.

### Staff roles and group structure

Sessions were facilitated by two qualified professionals, each with more than two years of clinical experience in Re‐Work support and SST. Facilitators were licensed professionals, including nurses, certified psychologists, and occupational therapists. Following the program manual, they operated the VR equipment and facilitated the group process.

Facilitators monitored engagement and safety, provided structured feedback, and supported rehearsal of interpersonal responses. To reduce assessment bias, staff involved in delivering SST‐VR did not conduct outcome assessments. Sessions typically included participants using VR headsets while others viewed the same scenario on a monitor, which supported discussion of strategies based on a common scenario. Supporting Information S1: Figure 1 provides an overview of SST‐VR and examples of session activities.

### Assessments

Before SST‐VR, the following baseline variables were collected: imipramine‐equivalent antidepressant dose, employment history, illness duration, and the Autism‐Spectrum Quotient, Japanese version (AQ‐J). For pre–post comparisons, participants completed the following self‐report measures: Kikuchi's Scale of Social Skills–18 items (KiSS‐18), the Social Adaptation Self‐evaluation Scale, Japanese version (SASS‐J), the Liebowitz Social Anxiety Scale, Japanese version (LSAS‐J), and the Beck Depression Inventory–Second Edition (BDI‐II).

All outcomes in this feasibility pilot were assessed using self‐report questionnaires. Objective vocational outcomes (e.g., return‐to‐work status, job retention, and absenteeism) and observer‐rated or performance‐based assessments of social functioning were not collected. All measures are standardized instruments with established reliability and validity in Japanese samples. Given the pilot sample size and study aims, internal consistency was not estimated in the present cohort.

### AQ‐J

The AQ is a 50‐item self‐administered questionnaire developed by Baron‐Cohen and colleagues to quantify autistic traits in adults across five domains: social skills, attention switching, attention to detail, communication, and imagination.^28^ The Japanese version (AQ‐J) was translated and standardized by Wakabayashi and colleagues and by Kurita and colleagues.^29^, ^30^ Prior studies have supported its internal consistency, factor structure, and discriminant validity in Japanese samples.^29^, ^30^ The AQ‐J is widely used as a screening and trait‐quantification measure in research and clinical settings, but it is not intended to establish a diagnosis on its own.

### KiSS‐18

The KiSS‐18 is a widely used instrument for assessing social skills relevant to interpersonal relationships.^31^ It was developed based on the Skills training framework by Goldstein and colleagues.^32^ The original framework categorizes social skills into six domains: basic skills, advanced skills, skills for dealing with emotions, skills as alternatives to aggression, skills for coping with stress, and planning skills.

The KiSS‐18 measures the degree to which these skills have been acquired. Each subscale corresponds to the following items:

Subscale 1 (Basic skills): Q1, Q5, Q15
Subscale 2 (Advanced skills): Q2, Q10, Q16
Subscale 3 (Skills for dealing with emotions): Q4, Q7, Q13
Subscale 4 (Skills as alternatives to aggression): Q3, Q6, Q8
Subscale 5 (Skills for coping with stress): Q11, Q14, Q17
Subscale 6 (Planning skills): Q9, Q12, Q18

Each item is rated on a 5‐point Likert scale. Total scores are calculated by summing all 18 items, with higher scores indicating greater overall social skills.

### SASS‐J

The Social Adaptation Self‐evaluation Scale (SASS) is a 21‐item self‐report questionnaire developed to assess social motivation and behavior, particularly in depression.^33^ It evaluates perceived social functioning across domains such as work, interpersonal relationships, and leisure activities. The SASS has been reported to be sensitive to change and to have acceptable reliability and validity.^34^ The Japanese version (SASS‐J) was translated and validated by Ueda and colleagues.^35^

### LSAS‐J

The Liebowitz Social Anxiety Scale (LSAS) assesses the severity of social anxiety by measuring fear and avoidance across social interaction and performance situations.^36^ In this study, the Japanese version (LSAS‐J) was administered as a self‐report measure. Sugawara and colleagues reported high internal consistency and supported structural validity in Japanese samples.^37^

### BDI‐II

The Beck Depression Inventory (BDI) was originally developed as a self‐report measure of depressive symptom severity.^38^ The revised version, BDI‐II, aligns item content with DSM criteria for major depressive disorder.^39^ It consists of 21 items rated on a 0–3 scale and has demonstrated acceptable psychometric properties across diverse populations.^40^, ^41^, ^42^

### Statistical analysis

All analyses were conducted using JASP (Version 0.95; JASP Team, 2025).^43^ The primary endpoint was the total score of Kikuchi's Social Skills Scale–18 (KiSS‐18). Within‐participant change scores (post–pre) were evaluated for approximate normality using the Shapiro–Wilk test and Q–Q plots. When assumptions were met, paired‐samples *t*‐tests were performed, and Cohen's *d* with 95% confidence intervals (CIs) was reported. Otherwise, the Wilcoxon signed‐rank test was used, and the rank‐biserial correlation (r_rb) with 95% CIs was reported.

To account for repeated measures and to explore moderation by baseline traits, we fitted a linear mixed‐effects model with fixed effects of Time (pre, post), mean‐centered baseline AQ total score, and their interaction, and a random intercept for participant. Models were estimated using restricted maximum likelihood. Time was coded as pre = 1 and post = 0; therefore, a negative coefficient for Time indicates higher post‐intervention means. Fixed effects were evaluated using likelihood ratio tests for nested models and are reported as *χ*² statistics with corresponding *p* values. Estimated marginal means at representative AQ values (mean and ±1 SD) were extracted for visualization. As a complementary analysis, we examined the association between baseline AQ and KiSS‐18 change scores while adjusting for baseline KiSS‐18.

Secondary outcomes (SASS‐J, LSAS‐J, and BDI‐II) and KiSS‐18 subscales were analyzed analogously. To control multiplicity for the three secondary outcomes, the Benjamini–Hochberg false discovery rate (FDR) procedure was applied across these tests (*m* = 3), and Supporting Information S1: Table 1 reports q values alongside unadjusted *p* values. Subscale analyses were exploratory and were not adjusted for multiplicity; therefore, these findings should be interpreted as hypothesis‐generating.

Sensitivity analyses used nonparametric bootstrap procedures with 2000 resamples to obtain bias‐corrected 95% CIs and bootstrap‐based *p* values for fixed effects, given the small sample size. All tests were two‐tailed with *α* = 0.05, and effect sizes with 95% CIs were reported for all outcomes regardless of statistical significance.

## RESULTS

### Participants

Of the 26 individuals assessed for eligibility, six were excluded because they did not meet the DSM‐5 diagnostic criteria for major depressive disorder (reasons summarized in Figure 1). The remaining 20 participants were enrolled, completed all six SST‐VR sessions and the post‐intervention assessment, and were included in the final analysis (Figure 1).

### Baseline characteristics

Participants had a mean age of 43.5 years (SD = 8.9); 18 were male and two were female. The mean duration of illness was 73.3 months (SD = 65.2), and the mean employment history was 20.2 years (SD = 9.4). Attendance was high, with a mean rate of 94.2% (SD = 9.8) for SST‐VR and 90.9% (SD = 11.1) for the Re‐Work program. No adverse events (e.g., cybersickness, dizziness, or increased anxiety) were reported during SST‐VR sessions, which was consistent with acceptable tolerability in this pilot.

At baseline, the mean KiSS‐18 total score was 49.7 (SD = 10.6). The mean AQ total score was 24.5 (SD = 8.0), with scores ranging from 8 to 40, indicating a restricted representation of very high autistic‐trait levels in this cohort. The AQ subscale means (SD) were as follows: social skills, 5.9 (2.5); attention switching, 5.8 (2.1); attention to detail, 4.0 (1.9); communication, 4.3 (2.5); and imagination, 4.7 (2.1). Additional baseline mean scores were 30.7 (SD = 7.6) for the SASS‐J, 52.7 (SD = 22.3) for the LSAS‐J, and 14.1 (SD = 8.3) for the BDI‐II. Most participants were receiving pharmacotherapy, and some had comorbid psychiatric disorders (e.g., anxiety disorders). None met criteria for bipolar spectrum, psychotic, or alcohol‐/substance‐use disorders based on the exclusion criteria.

### Primary outcome

**Figure 2:**
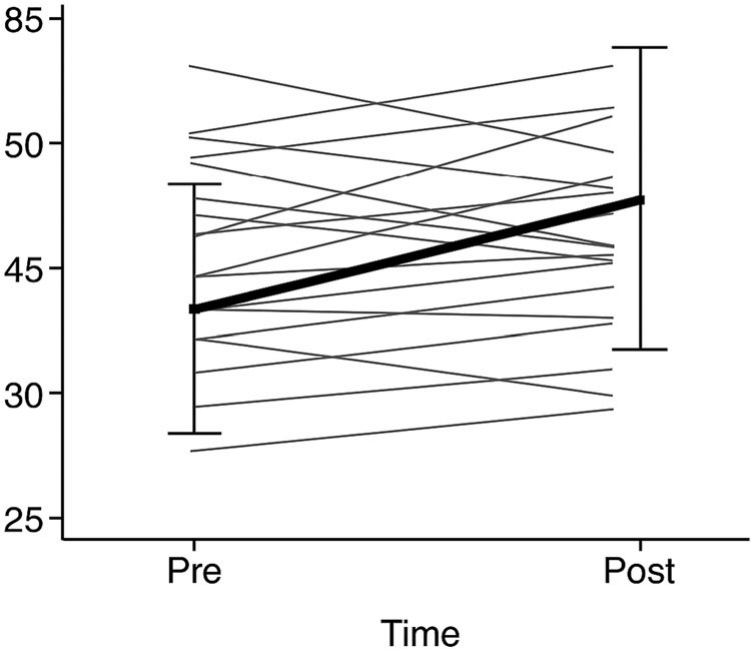
Individual KiSS‐18 total scores at baseline and postintervention. Lines connect participant‐level scores.

The KiSS‐18 total score increased from 49.7 ± 10.6 at baseline to 53.5 ± 12.4 postintervention (Figure 2). Because change scores (post–pre) deviated from normality, we used the Wilcoxon signed‐rank test, which indicated an increase in KiSS‐18 total scores (W = 46, *z* = −2.20, *p* = 0.028). The rank‐biserial correlation was r_rb = 0.562 (95% CI: −0.135 to 0.813), suggesting a moderate effect‐size estimate with substantial imprecision in this small pilot sample.

To explore moderation by baseline autistic traits while accounting for repeated measures, we fitted a linear mixed‐effects model with fixed effects of Time (pre/post), mean‐centered baseline AQ total score, and their interaction (Time × AQ), and a random intercept for participant. The model showed effects of Time (*χ*²(1) = 8.11, *p* = 0.004), baseline AQ (*χ*²(1) = 10.02, *p* = 0.002), and the Time × AQ interaction (*χ*²(1) = 4.46, *p* = 0.035). Bootstrap sensitivity analyses yielded similar inferences (Time: *p* = 0.009; AQ: *p* = 0.032; Time × AQ: *p* = 0.043).

Given the coding of Time (pre = 1, post = 0), a negative Time coefficient indicates higher post‐intervention means. Fixed‐effect estimates were consistent with an overall pre–post increase in KiSS‐18 (*β* = −5.82, SE = 1.84, *p* = 0.005) and lower overall KiSS‐18 levels at higher AQ (*β* = −0.88, SE = 0.24, *p* = 0.002). The positive Time × AQ term (*β* = 0.16, SE = 0.07, *p* = 0.037) suggested attenuated pre–post gains at higher AQ. Because the AQ range was restricted and the study was not powered for moderation testing, this interaction finding should be interpreted as exploratory.

**Figure 3:**
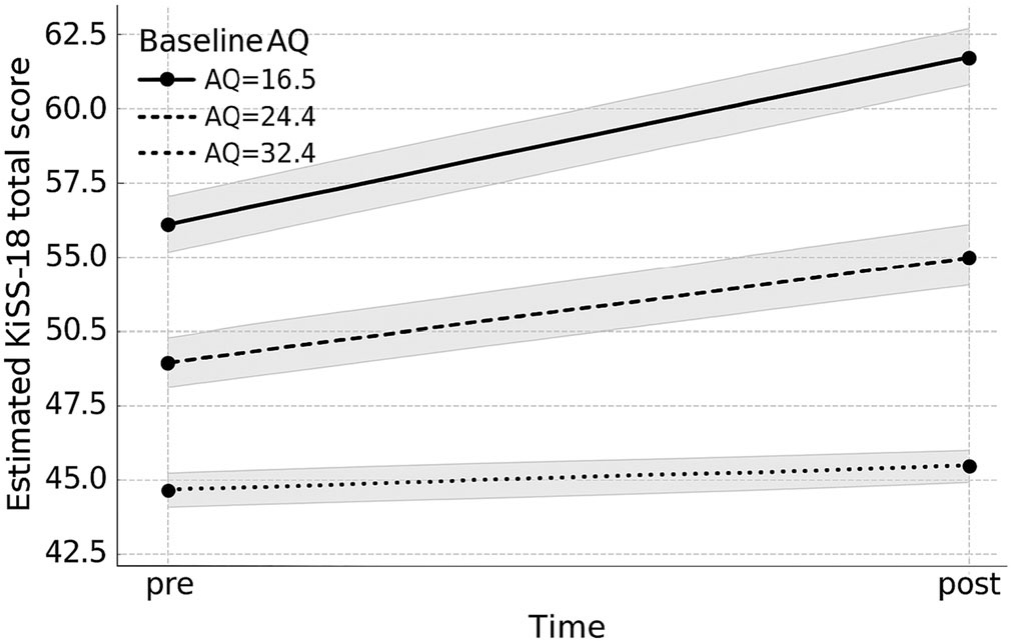
Changes in KiSS‐18 total scores across baseline AQ levels. Estimated marginal means of KiSS‐18 total scores at baseline and post‐intervention from the linear mixed‐effects model, evaluated at representative baseline Autism‐Spectrum Quotient (AQ) values (low = 16.5, mean = 24.4, high = 32.4; mean ± 1 SD). Shaded areas represent 95% confidence intervals. The Time × AQ pattern is exploratory.

Estimated marginal means at representative AQ values (mean ± 1 SD) illustrated this pattern: in the low‐AQ group, KiSS‐18 increased from 55.36 to 61.70; in the average‐AQ group, from 49.70 to 53.50; and in the high‐AQ group, from 44.04 to 45.30 (Figure 3).

**Figure 4:**
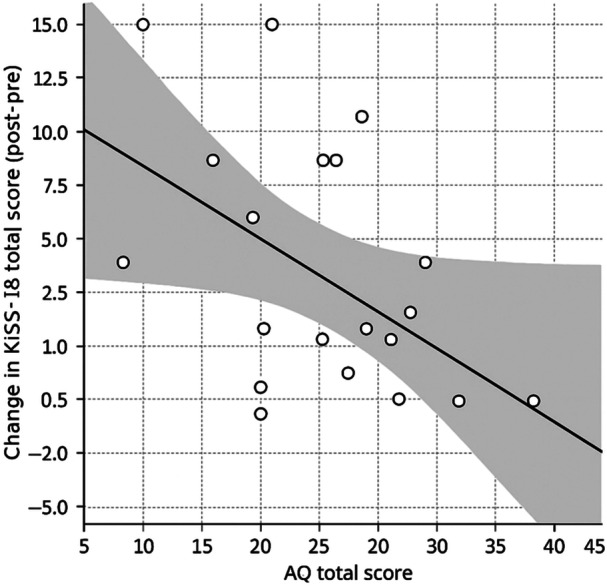
Relationship between baseline AQ and pre–post change in KiSS‐18 total scores. Scatterplot showing baseline Autism‐Spectrum Quotient (AQ) total score and change in KiSS‐18 total score (post–pre). The solid line indicates the fitted regression line, and the shaded area represents the 95% confidence interval. This analysis is exploratory.

As a complementary exploratory analysis, we regressed KiSS‐18 change scores on baseline AQ while adjusting for baseline KiSS‐18. The overall model fit was modest (*F*(2, 17) = 2.73, *p* = 0.094). Within this model, higher baseline AQ was associated with a smaller pre–post change in KiSS‐18 (*β* = −0.42, SE = 0.18, *p* = 0.034), whereas baseline KiSS‐18 was not (*p* = 0.340; Figure 4).

### Subscale analysis

**Figure 5:**
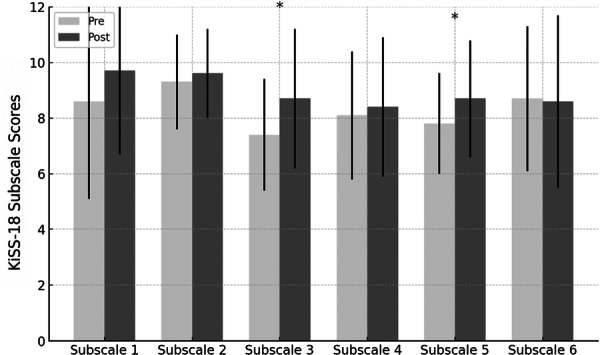
Pre–post changes in KiSS‐18 subscale scores. Subscale 1: Basic skills; Subscale 2: Advanced skills; Subscale 3: Emotional processing; Subscale 4: Skills alternative to aggression; Subscale 5: Stress management; Subscale 6: Planning skills. Bars represent means, and error bars indicate standard deviations (*n* = 20). Asterisks indicate unadjusted *p* < 0.05 from Wilcoxon signed‐rank tests (two‐tailed, *α* = 0.05). Subscale analyses were exploratory and not adjusted for multiplicity.

In exploratory analyses of KiSS‐18 subscales, post‐intervention scores were higher than baseline scores for the emotional processing and stress management subscales (Figure 5). Because these subscale analyses were not adjusted for multiplicity, they should be interpreted as hypothesis‐generating.

### Secondary outcomes

No significant pre–post changes were observed for the SASS‐J, LSAS‐J, or BDI‐II (Supporting Information S1: Table 1). After Benjamini–Hochberg FDR correction across these three outcomes, all q values were 0.133.

### Qualitative observations (supplementary)

These descriptive observations are provided in the Supplement to help contextualize the quantitative findings. We reviewed participants' free‐text comments collected immediately after SST‐VR sessions and summarized recurring topics, noting potential differences by baseline AQ subgroup.

Participants with lower AQ scores tended to highlight the perceived realism of the VR scenarios and described concrete interpersonal strategies they could apply at work, such as demonstrating empathy, appropriate self‐disclosure, and modulating prosody to support smoother conversations. They also frequently mentioned proactive coping behaviors, including asking questions promptly and using strategies to prevent conflict escalation.

In contrast, participants with higher AQ scores more often commented on self‐monitoring and meta‐communication processes. Their responses emphasized shifting attention from self to the interaction partner, managing the emotional impact of feedback, and coordinating verbal with nonverbal expression. Several participants also noted the substantial cognitive and emotional effort required to implement these skills in real time.

Overall, these patterns may be consistent with the exploratory Time × AQ interaction observed in the quantitative analyses, suggesting that baseline autistic traits could influence how participants engage with SST‐VR. Because these free‐text observations were not derived from a pre‐specified qualitative framework and were based on a small pilot sample, they should be interpreted as hypothesis‐generating and used to inform future controlled studies and more formal qualitative work.

## DISCUSSION

This single‐arm, add‐on feasibility pilot integrated SST‐VR into a psychiatric day‐care return‐to‐work program for adults with MDD. Attendance was high and no participants dropped out, supporting the practicality of delivering SST‐VR in a routine service setting. KiSS‐18 total scores were higher after the program than at baseline, with the largest gains observed in emotional processing and stress management. These patterns are broadly consistent with prior work on VR‐based SST. However, because we did not include a control condition and delivered SST‐VR alongside usual Re‐Work components, the observed pre–post changes cannot be attributed specifically to SST‐VR. Regression to the mean, increased contact and group engagement, and novelty or Hawthorne effects related to VR may also have contributed.^15^, ^22^, ^23^, ^44^

Secondary outcomes (SASS‐J, LSAS‐J, and BDI‐II) did not show significant pre–post change. One plausible explanation is timing. Broader domains such as social adaptation, social anxiety, and depressive symptoms often evolve more slowly than perceived skills and may require sustained practice and consolidation beyond the intervention period. Limited power in this feasibility sample further constrains inference. Future studies should incorporate longer follow‐up to evaluate downstream effects as participants re‐enter workplace contexts.

From an implementation standpoint, SST‐VR offers standardized, scenario‐based materials that may reduce session‐to‐session variability related to facilitator experience and staffing. We did not evaluate implementation outcomes such as fidelity or facilitator effects, so any delivery advantages remain provisional. Even so, the ability to replay identical scenarios may support safe trial‐and‐error practice and more structured reflection than conventional role‐play.^15^, ^24^ Future work should test these implementation features directly and examine whether they mediate clinical change.

SST‐VR in our setting also allowed participants to engage with the same scenarios either through a headset or via monitor‐based observation, providing a shared reference point for discussion. Prior work has described this “shared‐context” feature of VR‐based learning.^19^, ^20^ In this pilot, facilitators' informal notes suggested that participants differed in what they took from the sessions, with some emphasizing concrete behavioral tactics and others focusing on self‐monitoring and responses to feedback. Because these impressions were not systematically assessed, they should be viewed as descriptive and hypothesis‐generating.

The Time × AQ moderation finding is of interest but should be interpreted as exploratory. The analysis was underpowered for interaction effects, parameter estimates may be unstable, and the AQ range was restricted, limiting inference for individuals with higher autistic‐trait profiles or ASD‐level traits. One plausible explanation is that higher autistic traits increase cognitive load related to social cue recognition, attentional shifting, and integration of nonverbal information, which could constrain short‐term gains during a brief intervention.^45^, ^46^, ^47^ If this pattern replicates, participants with higher AQ may benefit from adaptations such as additional sessions, more explicit coaching on nonverbal communication, and stepwise rehearsal with structured feedback. These approaches should be tested prospectively in adequately powered controlled studies.

Several limitations should be emphasized. First, the single‐arm add‐on design without a control condition limits internal validity, and SST‐VR–specific effects cannot be separated from concurrent care or nonspecific influences. Second, this small sample from a single service setting was predominantly male and heterogeneous in illness duration, yielding imprecise estimates and limiting subgroup analyses. Third, outcomes were self‐reported; we did not include observer‐rated or performance‐based measures, nor objective vocational outcomes such as return‐to‐work status, absenteeism, or job retention. Fourth, implementation processes, including fidelity and facilitator effects, were not formally evaluated, and follow‐up was short. Future trials should compare Re‐Work plus SST‐VR against Re‐Work alone, include longer follow‐up, incorporate multimodal outcomes, and prespecify assessment of fidelity, facilitator effects, mechanisms of change, and moderation by autistic traits.

## CONCLUSION

This feasibility pilot suggests that SST‐VR can be integrated into a return‐to‐work program for adults with MDD, as indicated by high attendance and zero attrition. KiSS‐18 self‐reported social skills were higher postintervention than preintervention. However, because this single‐arm add‐on design cannot isolate SST‐VR‐specific effects from concurrent Re‐Work components and nonspecific influences such as regression to the mean, increased staff contact and group engagement, and novelty/Hawthorne effects, the KiSS‐18 change should be interpreted as hypothesis‐generating rather than evidence of efficacy. Outcomes were limited to self‐report measures; therefore, objective improvements in real‐world social functioning or vocational outcomes cannot be inferred. Generalizability is limited by the small, predominantly male sample and heterogeneity in illness duration. The Time × AQ interaction was exploratory and may be unstable given the small sample and restricted AQ range. Larger controlled studies, including comparisons with Re‐Work alone and longer follow‐up, are needed to test efficacy and clarify SST‐VR–specific contributions. Future trials should also prospectively evaluate AQ‐informed adaptations, such as additional sessions and more explicit coaching on nonverbal communication and feedback.

## AUTHOR CONTRIBUTIONS

Yutaro Akaki: Conceptualization and study design; data collection; manuscript drafting; supervision; final approval of the manuscript. **Yasuhisa Nakamura**: Conceptualization and study design; data analysis and interpretation; critical revision of the manuscript; final approval of the manuscript. **Yuko Nakanishi**: Conceptualization and study design; data collection; final approval of the manuscript. **Yuji Okuyama**: Conceptualization and study design; supervision; final approval of the manuscript. **Kiyoshi Fujita**: Critical revision of the manuscript; supervision; final approval of the manuscript. All authors have read and approved the final version of the manuscript.

## CONFLICT OF INTEREST STATEMENT

The authors declare no conflicts of interest. The FACEDUO VR program used in this study was provided by Otsuka Pharmaceutical Co., Ltd. and Jolly Good Inc. However, these companies had no involvement in the study design; data collection, analysis, or interpretation; manuscript preparation; or the decision to submit the manuscript for publication.

## ETHICS STATEMENT

This study was approved by the Ethics Committee of Okehazama Hospital, Fujita Kokoro Care Center (Approval No. R06‐026).

## PATIENT CONSENT STATEMENT

Written informed consent was obtained from all study participants.

## CLINICAL TRIAL REGISTRATION

Not applicable.

## Supporting information

SST‐VR Supplementary figure table.

## Data Availability

Raw data were generated at Fujita Mental Care Satellite Tokushige‐Kita Rework Center. Data supporting this study's findings are available from the corresponding author, Yasuhisa Nakamura, upon reasonable request.
