# Supplementary material for: Embedding virtual reality social skills training into return‐to‐work care for depression: A single‐arm feasibility pilot with exploratory autistic‐trait moderation
Source: PCN Rep. 2026 Feb 2;5(1):e70289. doi: 10.1002/pcn5.70289 (PMC12862656; doi:10.1002/pcn5.70289)
Supplement: Supplementary file 1 — SST‐VR Supplementary figure table. [file PCN5-5-e70289-s001.docx]

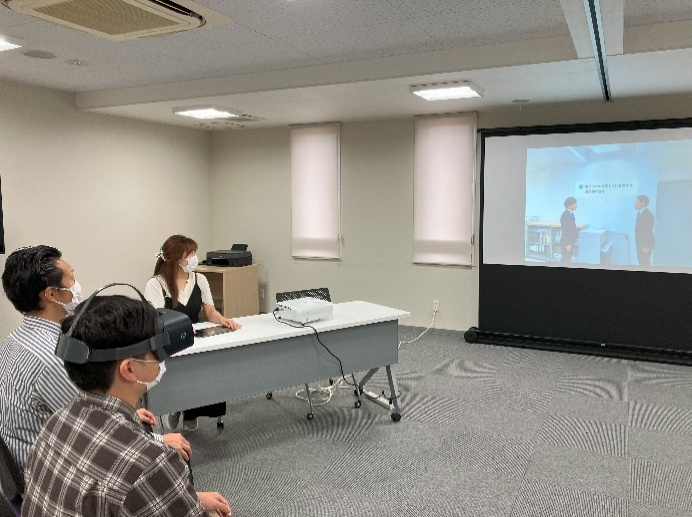

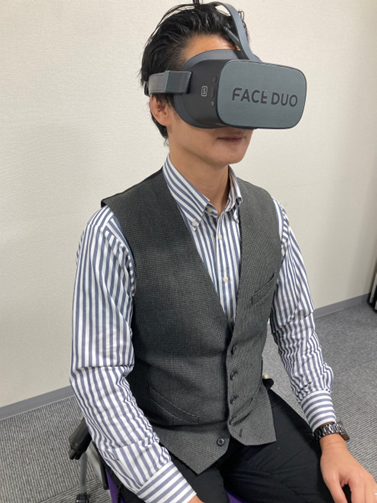


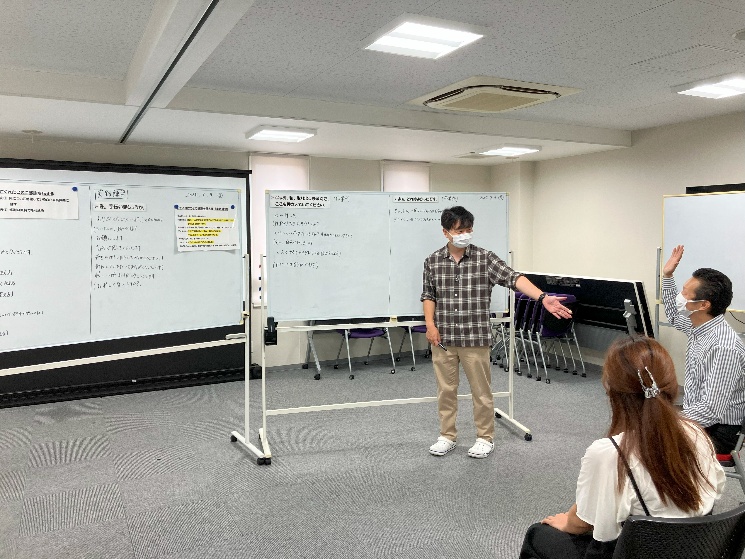


**
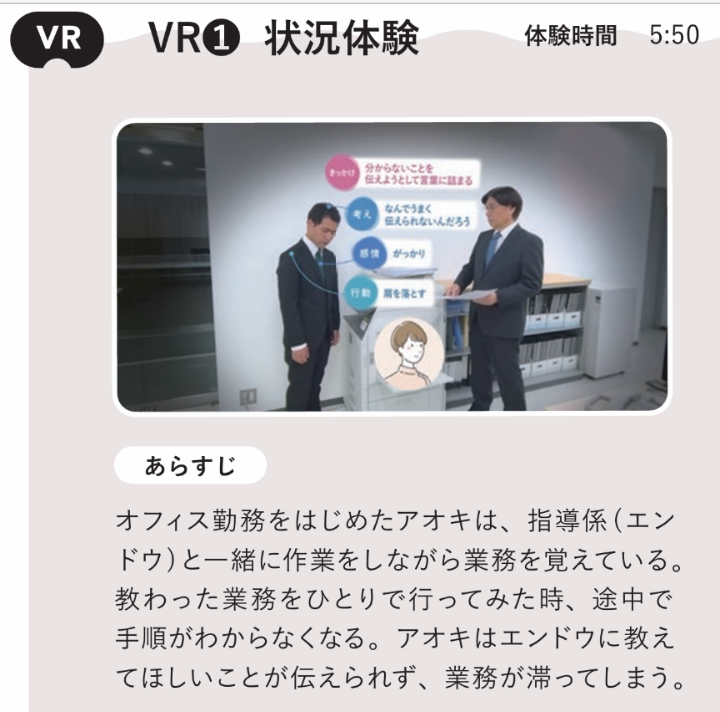
**

1. A participant wearing a VR headset. (b) A small-group SST-VR session in a psychiatric

day care setting.

**Supplementary Figure 1. SST-VR implementation based on six workplace-relevant interpersonal tasks.**

The SST-VR intervention comprised six biweekly sessions, each lasting approximately 90 minutes and conducted in small groups of 4–10 participants. Session content was derived from six interpersonal tasks adapted from the FACEDUO program, selected for their relevance to return-to-work challenges:

1. Making small talk during break time

2. Asking questions when uncertain about tasks

3. Avoiding sources of conflict

4. Responding to workplace feedback or warnings

5. Expressing gratitude for support

6. Making requests

Each session was structured into three parts: (i) situation experience, (ii) strategy discovery, and (iii) practical rehearsal. Training was delivered via immersive VR or monitor-based video viewing, with all participants experiencing direct VR headset use at least once during the intervention.

Abbreviations: SST-VR, social skills training using virtual reality.

**Supplementary Table 1. Changes in Secondary Outcomes**

| outcome | Pre | Post | Test statistic | *p*-value | FDR  *q*-value | Effect size (95% CI) |
| --- | --- | --- | --- | --- | --- | --- |
| SASS-J | 30.7 ± 7.6 | 32.8 ± 6.4 | Wilcoxon W = 52.0, z =－1.73 | 0.086 | 0.133 | r_rb = ―0.256 (−0.762 to 0.025) |
| LSAS-J | 52.7 ± 22.3 | 31.4 ± 15.6 | Wilcoxon W=120.5, z =1.52 | 0.133 | 0.133 | r_rb = 0.262 (−0.091 to 0.745) |
| BDI-II | 14.1 ± 8.3 | 11.2 ± 5.5 | Wilcoxon W =137.5, z =1.71 | 0.091 | 0.133 | r_rb = 0.256 (−0.031 to 0.759) |

Benjamini–Hochberg false discovery rate (FDR) correction was applied across the three outcomes (m = 3).

Abbreviations: BDI-II, Beck Depression Inventory–Second Edition; CI, confidence interval; FDR, false discovery rate; LSAS-J, Liebowitz Social Anxiety Scale—Japanese Version; SASS-J, Social Adaptation Self-evaluation Scale—Japanese Version.
